# Endoscopic Management of Crayfish (*Procambarus clarkii*) Aspiration: A Case Report

**DOI:** 10.70352/scrj.cr.25-0444

**Published:** 2025-12-04

**Authors:** Guo-Qiang Song, Guo-Qiang Hu

**Affiliations:** Department of Respiratory, Changxing County Hospital of Traditional Chinese Medicine, Huzhou, Zhejiang, China

**Keywords:** airway foreign body, aphonia, vocal cord obstruction, flexible bronchoscopy, crayfish

## Abstract

**INTRODUCTION:**

Airway foreign bodies are time-sensitive otolaryngologic emergencies that can occur in persons of any age. We describe a unique case of complete aphonia caused by the impaction of a crayfish (*Procambarus clarkii*) claw at the level of the vocal cords.

**CASE PRESENTATION:**

A 65-year-old man developed abrupt throat discomfort and loss of voice while eating crayfish. Indirect laryngoscopy showed a foreign body resting across the vocal cords with absent vibration. Cervical CT demonstrated a tubular hyperdense structure within the glottic region, and flexible transoral bronchoscopy confirmed a mucus-coated object adherent to the vocal cords. The object was removed en bloc with grasping forceps under topical anesthesia, after which phonation returned immediately. The patient was discharged the next day without complications. The retrieved foreign body was identified as a crayfish claw.

**CONCLUSIONS:**

Crayfish claw impaction at the vocal cords is an exceedingly rare cause of aphonia. Incorporating a targeted dietary history—particularly of regional or seasonal foods—can expedite the diagnosis and management of unusual airway foreign bodies.

## INTRODUCTION

Airway foreign bodies remain a time-sensitive clinical emergency across all age groups and contribute to morbidity and mortality worldwide. The spectrum of aspirated materials is broad and includes coins, teeth, medical instruments, and region- or culture-specific foods. Prompt recognition is essential because delayed diagnosis increases the risk of airway compromise and pulmonary complications. We report, to our knowledge, one of the first documented cases of a crayfish (*Procambarus clarkii*) claw lodged across the vocal cords and producing complete aphonia. Given the widespread seasonal consumption of crayfish in many parts of China, this case underscores the importance of obtaining a detailed dietary history when evaluating unexplained hoarseness, aphonia, or suspected upper-airway obstruction.

## CASE PRESENTATION

A previously healthy 65-year-old man experienced sudden throat discomfort and complete loss of voice while eating a meal that included crayfish. He denied choking, dyspnea, dysphagia, or cough at symptom onset but reported persistent throat fullness.

On presentation to our department, indirect laryngoscopy revealed a foreign body spanning and impinging upon the true vocal cords, preventing their normal vibration. No active bleeding was observed.

**Fig. 1:**
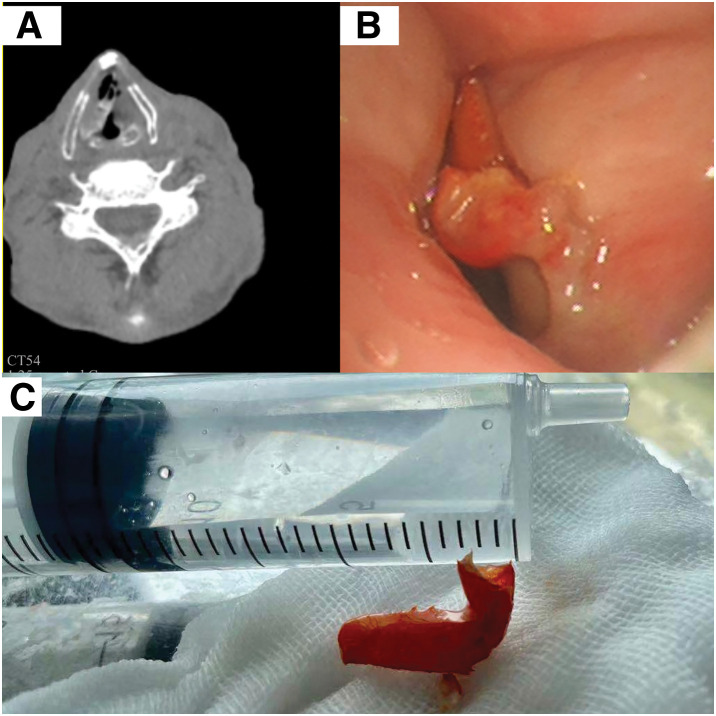
Crayfish claw lodged between the vocal cords, causing aphonia. (**A**) Cervical CT showing a tubular hyperdense lesion. (**B**) Flexible bronchoscopy demonstrating the claw between the vocal cords. (**C**) Retrieved crayfish claw.

Noncontrast cervical CT demonstrated a tubular hyperdense lesion in the glottic region (**Fig. 1A**), consistent with a possible exogenous foreign body.

After topical oropharyngeal anesthesia with lidocaine again (no systemic sedation), flexible transoral bronchoscopy was performed. A mucus-coated, chitinous foreign body was visualized firmly adherent between the vocal cords (**Fig. 1B**). Using Boston Scientific (Marlborough, MA, USA) Radial Jaw 4 Pulmonary Standard Capacity grasping forceps (2.0 mm jaw diameter, 1.8 mm minimum channel), the object was securely grasped. The patient was then instructed to exhale slowly and continuously to relax the laryngeal muscles and minimize spasm, after which the foreign body was rapidly removed in 1 piece with gentle manipulation to avoid laryngeal trauma or bleeding. Phonation returned as soon as the patient attempted to speak following removal. Repeat bronchoscopic inspection confirmed that no residual material remained. The patient was observed overnight, remained asymptomatic, and was discharged the following day. At 1-week follow-up, he reported sustained normal voice and no respiratory complaints.

Gross examination of the retrieved object revealed a 19-mm claw from a crayfish (*Procambarus clarkii*) (**Fig. 1C**), a highly popular seasonal delicacy in China.

## DISCUSSION

Foreign body aspiration and related airway foreign bodies affect both children and adults and represent an ongoing global public health concern. Incidence varies by region, age, and exposure patterns but remains substantial worldwide.^1)^ Dietary customs and food preparation practices influence the types of objects aspirated; items such as bones, nuts, shells, and other indigenous foods (e.g., fish bones, chicken bones, or jujube pits commonly reported in East Asia) feature prominently in many regional series.^2)^

A meticulous clinical history—including temporal relation to eating, witnessed aspiration, and food types—is critical for early diagnosis.^3)^ Imaging supports localization and assessment of complications.^4)^ High-resolution CT with airway reconstructions improves detection accuracy and can reduce negative bronchoscopies in selected patients, although clinical judgment remains paramount.^5)^ CT is also useful for confirming complete removal when visualization is limited or when symptoms persist after extraction.^6)^

Bronchoscopic retrieval remains the mainstay of treatment for airway foreign bodies.^7)^ The choice between flexible and rigid bronchoscopy depends on patient age, foreign-body type and location, operator expertise, and available resources.^8)^ Comparative studies suggest that flexible bronchoscopy can achieve high success rates in appropriately selected cases, whereas rigid bronchoscopy remains indispensable in many pediatric and complex scenarios.^9)^ According to existing evidence, employing an induction strategy that preserves spontaneous ventilation can reduce the risk of progression from partial to complete proximal airway obstruction.^10)^ Reported complication rates for bronchoscopy—when performed by experienced teams—are generally low.

### Unique presentation: isolated aphonia without dyspnea

A key aspect of this case is the presentation of complete aphonia without accompanying dyspnea or stridor, which contrasts with more typical lower-airway foreign bodies that often cause wheezing, cough, or respiratory distress. Vocal cord impaction can mechanically prevent cord vibration necessary for phonation, leading to isolated voice loss while preserving adequate airflow for breathing. This atypical manifestation has been reported in rare cases, such as coin impaction at the glottic level causing sudden aphonia due to accidental ingestion of a coin carried in the mouth, metallic spring foreign bodies resulting in aphonia initially misdiagnosed as vocal cord paralysis.^11,12)^ Physiologically, if the foreign body spans the glottis without fully occluding it, ventilation may remain intact, but vocalization is impaired due to disrupted cord approximation and vibration.^13)^ Such presentations risk misdiagnosis as functional aphonia, vocal cord paralysis, or psychogenic disorders, especially in the absence of respiratory symptoms.^14)^ Clinicians should prioritize early laryngoscopy in patients with sudden aphonia and a dietary history suggestive of aspiration, as delays can lead to complications such as edema or infection. In our case, the crayfish claw’s tubular shape likely allowed partial airflow, explaining the lack of dyspnea despite complete voice obstruction. This underscores the need for heightened awareness in regions with high crustacean consumption.

### Implications of the present case

Our patient’s crayfish claw lodged at the vocal cords caused immediate aphonia without significant lower-airway symptoms, highlighting that upper-airway foreign bodies may present with isolated voice changes rather than respiratory distress. Because crayfish consumption is common in many regions of China during spring and summer, clinicians should specifically inquire about recent ingestion of crustacean dishes when evaluating acute-onset hoarseness or aphonia. Early laryngoscopic examination and, when indicated, CT imaging can expedite definitive endoscopic removal and prevent airway compromise.

## CONCLUSIONS

Crayfish claw impaction at the vocal cords is a rare but potentially serious cause of aphonia, particularly in regions where seasonal crayfish dishes are popular, such as in East Asia. A thorough dietary history, prompt laryngoscopic evaluation, targeted CT imaging, and timely bronchoscopic extraction together enable rapid diagnosis and favorable outcomes. Increased awareness of region-specific foods as potential airway foreign bodies may help reduce diagnostic delays and associated complications.
